# Internet Hospitals Help Prevent and Control the Epidemic of COVID-19 in China: Multicenter User Profiling Study

**DOI:** 10.2196/18908

**Published:** 2020-04-14

**Authors:** Kai Gong, Zhong Xu, Zhefeng Cai, Yuxiu Chen, Zhanxiang Wang

**Affiliations:** 1 The Internet Hospital of the First Affiliated Hospital of Xia'men University Xia'men City China; 2 Zoenet Health Company Limited Xia'men City China

**Keywords:** internet hospital, telemedicine, novel coronavirus disease, pandemic, prevention, control, coronavirus, COVID-19, public health, infectious disease

## Abstract

**Background:**

During the spread of the novel coronavirus disease (COVID-19), internet hospitals in China were engaged with epidemic prevention and control, offering epidemic-related online services and medical support to the public.

**Objective:**

The aim of this study is to explore the role of internet hospitals during the prevention and control of the COVID-19 outbreak in China.

**Methods:**

Online epidemic-related consultations from multicenter internet hospitals in China during the COVID-19 epidemic were collected. The counselees were described and classified into seven type groups. Symptoms were recorded and compared with reported patients with COVID-19. Hypochondriacal suspicion and offline visit motivation were detected within each counselees’ group to evaluate the social panic of the epidemic along with the consequent medical-seeking behaviors. The counselees’ motivation and the doctors’ recommendation for an offline visit were compared. Risk factors affecting the counselees’ tendency of hypochondriacal suspicion and offline visit motivation were explored by logistic regression models. The epidemic prevention and control measures based on internet hospitals were listed, and the corresponding effects were discussed.

**Results:**

A total of 4913 consultations were enrolled for analysis with the median age of the counselees at 28 years (IQR 22-33 years). There were 104 (2.12%) healthy counselees, 147 (2.99%) hypochondriacal counselees, 34 (0.69%) exposed counselees, 853 (17.36%) mildly suspicious counselees, 42 (0.85%) moderately suspicious counselees, 3550 (72.26%) highly suspicious counselees, and 183 (3.72%) severely suspicious counselees. A total of 94.20% (n=4628) of counselees had epidemic-related symptoms with a distribution similar to those of COVID-19. The hypochondriacal suspicion (n=2167, 44.11%) was common. The counselees’ motivation and the doctors’ recommendation for offline visits were inconsistent (*P*<.001) with a Cohen kappa score of 0.039, indicating improper medical-seeking behaviors. Adult counselees (odds ratio [OR]=1.816, *P*<.001) with epidemiological exposure (OR 7.568, *P*<.001), shortness of breath (OR 1.440, *P*=.001), diarrhea (OR 1.272, *P*=.04), and unrelated symptoms (OR 1.509, *P*<.001) were more likely to have hypochondriacal suspicion. Counselees with severe illnesses (OR 2.303, *P*<.001), fever (OR 1.660, *P*<.001), epidemiological exposure history (OR 1.440, *P*=.01), and hypochondriacal suspicion (OR 4.826, *P*<.001) were more likely to attempt an offline visit. Reattending counselees (OR 0.545, *P*=.002) were less motivated to go to the offline clinic.

**Conclusions:**

Internet hospitals can serve different types of epidemic counselees, offer essential medical supports to the public during the COVID-19 outbreak, reduce the social panic, promote social distancing, enhance the public’s ability of self-protection, correct improper medical-seeking behaviors, reduce the chance of nosocomial cross-infection, and facilitate epidemiological screening, thus, playing an important role on preventing and controlling COVID-19.

## Introduction

From late 2019 to early 2020, an outbreak of novel coronavirus disease (COVID-19) spread throughout China and soon became a global concern [1-3]. The Chinese government had adopted a series of administrative measures to stop the spread of the epidemic [4], including promulgating decrees that required the domestic internet hospitals to vigorously carry out remote medical services in response to the epidemic [5]. Under such circumstances, the internet hospitals in China, which is a new approach to outpatient health care that provides health services via internet technologies [6], were engaged in the epidemic prevention and control, opening up free COVID-19 consultation services as the main form of remote medical services for the public during the epidemic. This is the first time that internet hospitals have been involved in the response to an infectious public health incident. Its role during the epidemic prevention and control has yet to be explored. This study analyzed the details of the free epidemic consultations from multicenter internet hospitals in China during the COVID-19 outbreak. Through user profiling, we assessed the social panic and the public’s medical needs during the COVID-19 outbreak, revealed the effects of internet hospitals on the epidemic prevention and control, and expounded and explored the managing strategies to make the internet hospitals play a greater role in the infectious public health emergency responses.

## Methods

### Data Sources

We collected 8913 consecutive deidentified free online consultations generated between January 25, 2020, and February 25, 2020, from 30 general public internet hospitals in 11 provinces of China outside of the Hubei area. The consultants were certified doctors from the general public hospitals, and the counselees were local residents who were not admitted to the offline hospitals and supposed to have epidemic-related questions. All of the data were extracted from the platform of Zoenet Health Company Limited [7], which cooperates with public hospitals to run the online medical services. A total of 405 invalid consultations containing duplicated or nonmedical contents were removed. Through semantic analysis, 563 repetitive and 3032 unrelated consultations, in which the counselees had no related symptoms and asked questions irrelevant to the epidemic, were also excluded. Each of the remaining consultations were analyzed artificially by one trained researcher and rechecked by another. Any divergence from this procedure was resolved by discussion with all researchers.

### Study Definitions

Variables including age, sex, symptoms, reattendance, epidemiological exposure history, hypochondriacal suspicion, offline visit recommendation, and offline visit motivation were recorded. The epidemic-related symptoms were classified into common and uncommon epidemic symptoms. Fever (axillary temperature of 37.5°C or higher), cough, expectoration, myalgia, and fatigue were classified into common symptoms from which most of the patients with COVID-19 outside of Wuhan in China suffered from as described by Xiao-Wei Xu et al [8]. Symptoms including mild fever (axillary temperature between 37°C and 37.5°C), nasal congestion, headache, sore throat, shortness of breath, diarrhea, chills, nausea, and vomiting were recognized as uncommon epidemic symptoms that may potentially be caused by COVID-19 [9-11]. Other symptoms (eg, palpitation, dizziness, unexplained abdominal pain, eye discomfort) were also recorded and categorized as unrelated symptoms. Reattendance means the counselees had once consulted doctors either through online or offline approaches before the current consultation. Epidemiological exposure refers to the history of travel or residence in Wuhan and surrounding areas within the Hubei province or other communities with case reports, as well as the history of contact with patients with COVID-19 or contact with people with epidemic-related symptoms from Wuhan and surrounding areas or from communities with case reports. All of the above epidemiological exposure should happen within 14 days before the onset of illness. Hypochondriacal suspicion is whether the counselees had clearly expressed their concern of being potentially infected by the novel coronavirus. Offline visit recommendation means whether the online doctors had suggested an offline consultation. The counselees who had received offline visit recommendations were considered as being in severe condition. Offline visit motivation means whether the counselees had expressed their attempts to go to the offline clinics.

**Figure 1:**
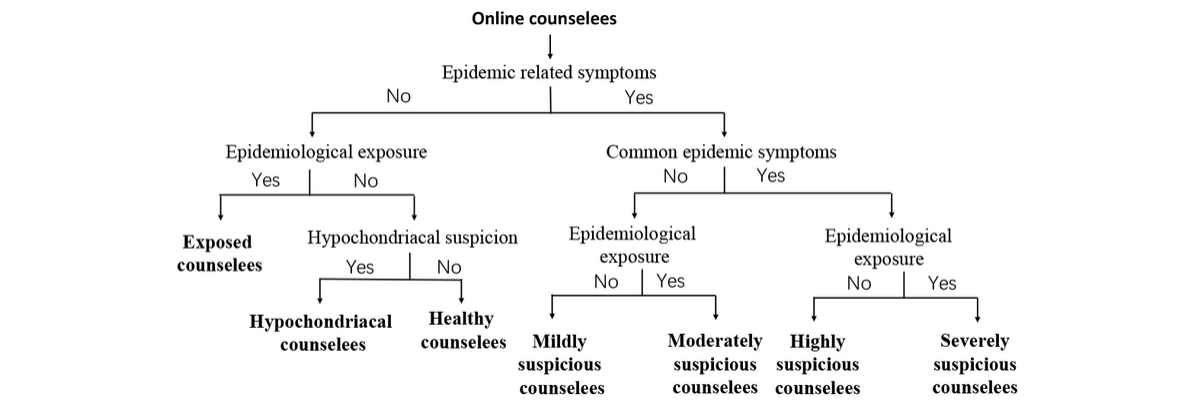
Classification of different counselees.

We classified the counselees into seven type groups, including healthy counselees, hypochondriacal counselees, exposed counselees, mildly suspicious counselees, moderately suspicious counselees, highly suspicious counselees, and severely suspicious counselees (Figure 1). The healthy counselees had neither epidemic-related symptoms nor any epidemiological exposure history, simply asking epidemic-related questions without hypochondriacal suspicion. The hypochondriacal counselees had hypochondriacal suspicion without any epidemic exposure nor any epidemic-related symptoms. The exposed counselees had epidemiological exposure history without epidemic-related symptoms. The mildly suspicious counselees had certain uncommon epidemic symptoms without any common epidemic symptoms nor epidemiological exposure history. The moderately suspicious counselees had both uncommon epidemic symptoms and epidemiological exposure history without any common epidemic symptoms. The highly suspicious counselees had common epidemic symptoms without epidemiological exposure history. The severely suspicious counselees had both common epidemic symptoms and epidemiological exposure history.

### Statistical Analysis

The amount and the percentage of counselees with positive hypochondriacal suspicion, offline visit motivation, and an offline visit recommendation were counted and calculated within different counselee groups. All symptoms were extracted and compared with patients with reported COVID-19 [11]. Categorical variables were compared using the chi-square test. The Cohen kappa score was calculated to assess the consistency of the counselees’ motivation and the doctors’ recommendation for offline visits.

Univariate and multivariate logistical regression analyses were conducted to predict the risk factors for hypochondriacal suspicion and offline visit motivation. Predictors that were statistically significant in the univariate analyses (*P*<.05) were included in multivariate logistic regression models, with odds ratios and CIs calculated. Epidemiological exposure history, adulthood, sex, severe illness condition, reattendance, and symptoms were considered as the predictors of hypochondriacal suspicion; all of the previously mentioned predictors, with the addition of hypochondriacal suspicion, were considered as the predictors of offline visit motivation.

Statistical analysis was done by Python (version 3.6; Python Software Foundation). The forest figure showing the results of logistic regressions was drawn by R (version 3.6.2; R Foundation for Statistical Computing). *P*<.05 was considered statistically significant.

### Ethics Statement

Ethic approval was obtained from the medical research ethics committee of the first affiliated hospital of Xiamen University, Xiamen, China (protocol number 3502Z2020YJ05) before the start of the study.

## Results

### Characteristics of the Counselees

A total of 4913 consultations were finally enrolled for analysis including 2031 (41.34%) males and 2882 (58.66%) females. The median age was 28 years (IQR 22-33 years). All children younger than 12 years had online consultations completed by their guardians. Epidemiological exposure history was reported for 259 (5.27%) counselees. Epidemic-related symptoms were reported for 4628 (94.20%) counselees, and 3733 (75.98%) had common-epidemic symptoms. Hypochondriacal suspicion was reported for 2165 (44.07%) counselees, and 869 (17.69%) were motivated to do an offline visit. A total of 190 (3.87%) were in severe condition with an affirmative offline visit recommendation. Only 2 severe cases had no epidemic-related symptoms, but both of them had hypochondriacal suspicion: a 39-year-old male who felt precordial discomfort and a 2-year-old girl who was found in a drowsy state by her parents. Most severe counselees were children less than 10 years old (102/190, 53.7%).

After classification of all the counselees, 104 (2.12%) were healthy counselees, 147 (2.99%) were hypochondriacal counselees, 34 (0.69%) were exposed counselees, 853 (17.36%) were mildly suspicious counselees, 42 (0.85%) were moderately suspicious counselees, 3550 (72.26%) were highly suspicious counselees, and 183 (3.72%) were severely suspicious counselees. Hypochondriacal suspicion and offline-visit motivation were common within different types of counselees. However, fewer counselees had received affirmative offline-visit recommendations (Table 1).

**Table 1:** Hypochondriacal suspicion, offline visit motivation, and offline visit recommendation in different counselees’ type groups.

Counselee type	Hypochondriacal suspicion, n (%)	Offline visit motivation, n (%)	Offline visit recommendation, n (%)
Healthy counselees, n=104	0 (0.0)	3 (2.9)	0 (0.0)
Hypochondriacal counselees, n=147	147 (100.0)	18 (12.2)	2 (1.4)
Exposed counselees, n=34	32 (94.1)	12 (35.3)	0 (0.0)
Mildly suspicious counselees, n=853	419 (49.1)	132 (15.5)	1 (0.1)
Moderately suspicious counselees, n=42	38 (90.5)	18 (42.9)	0 (0.0)
Highly suspicious counselees, n=3550	1378 (38.8)	635 (17.9)	185 (5.2)
Severely suspicious counselees, n=183	151 (82.5)	51 (27.9)	2 (1.1)
Total number, N=4913	2165 (44.07)	869 (17.69)	190 (3.87)

### Distribution of the Counselees’ Symptoms

**Figure 2:**
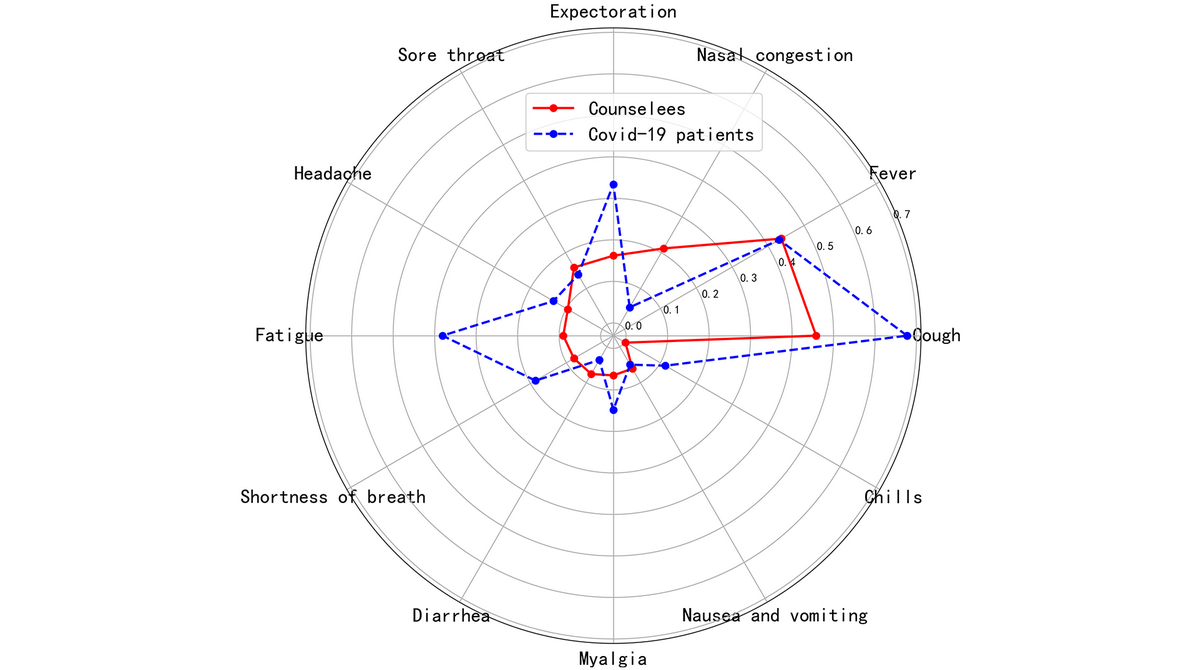
The symptom distribution of online counselees and COVID-19 patients. COVID-19: coronavirus disease.

For the 4628 counselees with epidemic-related symptoms, cough (n=2118, 45.76%) was the most common symptom, followed by fever (n=2021, 43.67%), nasal congestion (n=981, 21.20%), expectoration (n=752, 16.25%), sore throat (n=735, 15.88%), headache (n=443, 9.57%), fatigue (n=415, 8.97%), shortness of breath (n=365, 7.89%), diarrhea (n=350, 7.56%), myalgia (n=301, 6.50%), nausea and vomiting (n=282, 6.09%), and chills (n=16, 0.35%) (see Figure 2).

### Improper Medical-Seeking Behaviors

The counselees’ motivation and the doctors’ recommendation for offline visits were significantly different (χ^2^_1_=13.4230, *P*<.001) with a Cohen kappa score of 0.039. Of the 190 severe conditions, there were 137 (72.1%) counselees who did not attempt an offline visit for further treatment. Of the 4728 nonsevere cases, 816 (17.26%) counselees with mild conditions were motivated for offline medical care without an affirmative recommendation.

### Risk Factors for Hypochondriacal Suspicion and Offline Visit Motivation

**Figure 3:**
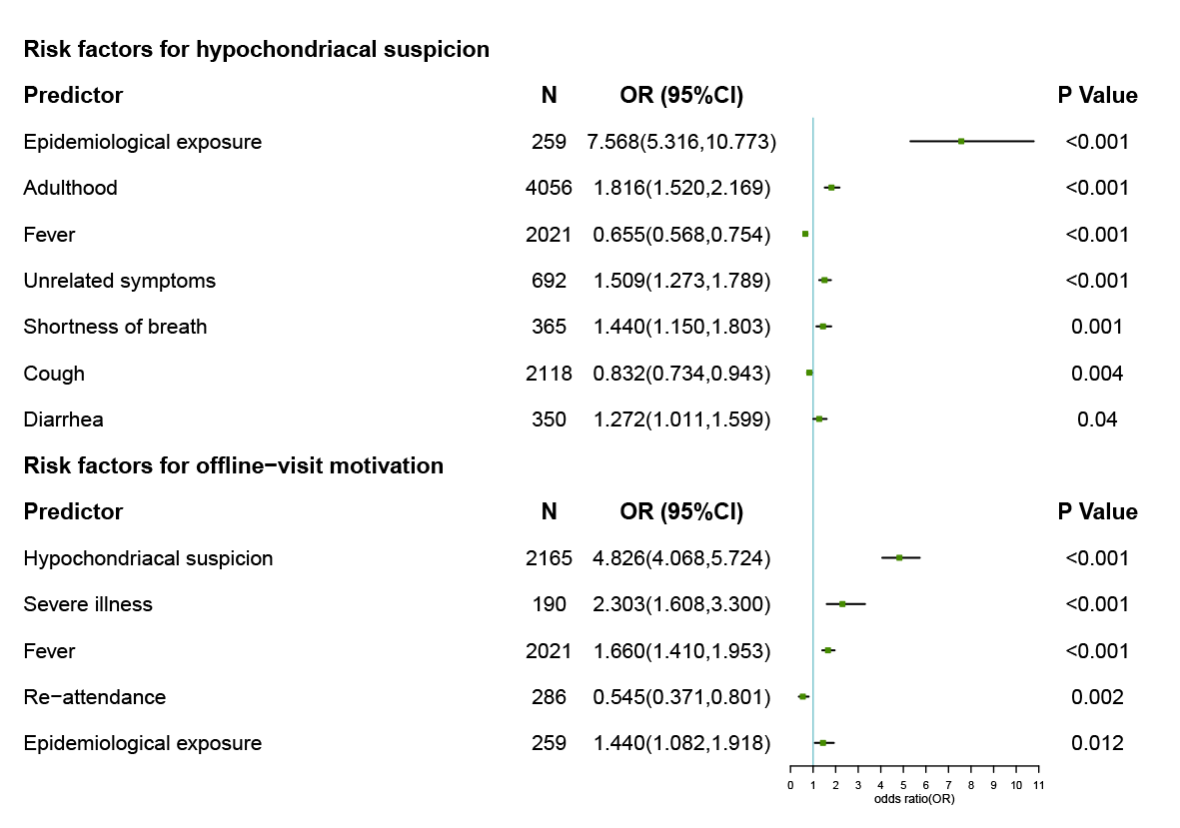
The independent risk factors for hypochondriacal suspicion and offline visit motivation.

The multivariate logistic regression models showed that epidemiological exposure, adulthood, shortness of breath, diarrhea, and unrelated symptoms can independently increase the probability of hypochondriacal suspicion; fever and cough can reduce the probability of hypochondriacal suspicion. Severe illness, fever, epidemiological exposure, and hypochondriacal suspicion can increase the probability of offline visit motivation; however, reattending counselees were less likely to have offline visit motivation (Figure 3).

## Discussion

### Principal Findings

Our results showed that the epidemic of COVID-19 brought panic and hypochondria to the public, further inducing improper health-seeking behaviors and increased demand of medical care services. Along with the arrival of information times, the traditional management style could not adapt to the public’s needs during the period of the COVID-19 outbreak. Internet hospitals may serve different types of epidemic counselees, helping prevent and control the epidemic of COVID-19 in China.

### Medical Contradictions Brought by COVID-19

As the epidemic of COVID-19 in China overlapped with the high incidence for common cold and seasonal influenza [12], many residents got certain respiratory or other symptoms similar to COVID-19 (Figure 2). A recent large epidemiological study found that physical symptoms (eg, myalgia, dizziness, coryza) and poor self-rated health statuses were significantly associated with a greater psychological impact of the COVID-19 outbreak and higher levels of stress, anxiety, and depression among Chinese individuals [13]. Our results showed that nearly half of the counselees had hypochondriacal suspicion; besides epidemiological exposure, both epidemic-related and unrelated symptoms can exacerbate hypochondria, which can encourage counselees’ unnecessary motivation for an offline visit. This is why a considerable proportion of counselees were motivated for an offline visit without the doctor’s recommendation. On the contrary, most of the counselees in severe conditions were reluctant to go to an offline clinic for fear of cross-infection. The counselees’ motivation was significantly different from the doctors’ recommendation, leading to improper health-seeking behaviors, bringing further harm to the public health.

Under the stress of the epidemic, an increasing number of people need professional medical guidance, but the offline hospital visits should be strictly restricted to cut off nosocomial transmission routes. The demanding medical services and the inaccessibility of medical care became one of outstanding contradictions during the COVID-19 outbreak. The internet hospitals may solve this dilemma by offering equitable and inclusive online services to assist the epidemic control [14].

### Internet Hospitals in China During the COVID-19 Epidemic

After recent years of development, the internet hospitals in China can now break through the limitations of time and space with excellent accessibility, providing a variety of medical services to all citizens [15]. Through internet connections, interdisciplinary and cross-regional collaborations can be accomplished to improve the capability of dealing with emerging diseases. The Chinese government had encouraged internet hospitals to join the epidemic prevention and control efforts at the beginning of the COVID-19 outbreak [5] and confirmed their role as one important part of the joint epidemic prevention and control system [16]. On March 15, 2020, the first professional standard, “Specification for online consultation service for infectious disease epidemic situation,” was published on the national group standard information platform of China [17], requiring that internet hospitals provide 24/7 online services in response to the epidemic, including prehospital services such as initial screening and medical education; intrahospital services such as offline service appointment and offline visit guidance; and posthospital services such as psychological counseling, post services for medical records, report interpretation for reattending patients, and drug delivery services for patients with chronic diseases. These services should cover all potential medical needs of the public during the epidemic. Meanwhile, the internet hospitals were required to establish intact traceable health files for each counselee and share the data with the supervising departments including the Centers for Disease Control and Prevention (CDC) and local health commissions.

### Effects of Internet Hospitals on the Epidemic Prevention and Control

Through the above measures, internet hospitals may help prevent and control the COVID-19 epidemic with both primary and auxiliary functions. In terms of primary functions, first, internet hospitals may reduce the crowd gatherings in offline hospitals through multiple approaches. Through online education and propaganda [18] as well as psychological interventions, the online medical services not only teach the public essential epidemic-protective skills, but also alleviate the social panic and help release the public’s hypochondriacal suspicions, thus, reducing the unnecessary offline hospital visits and enhancing psychological resilience [19]. This is consistent with our results that there was less offline visit motivation for the reattending counselees. Meanwhile, the various handy services provided by internet hospitals may reduce the patients’ repeated visits. These measures together may effectively reduce people gathering in offline hospitals [20]. Furthermore, internet hospitals run by public hospitals can connect the online services with the offline medical procedures, offering online triage services and guiding the counselees to the corresponding offline departments according to their symptoms [21,22]. This would make the suspected cases walk through isolated channels to the specialized outpatient clinic, thus, reducing people’s contact within hospitals and further reducing the chance of getting infected [8]. Furthermore, this protects our medical staff, who are the major force in confronting the epidemic [23], from a massive offline workload and unnecessary occupational exposure [24,25]. Second, the internet hospitals may play a greater role through the integration of online resources and offline epidemic control efforts. As most of the provinces in China have initiated a level-1 public health response to control COVID-19, a joint prevention and control system with online information and an offline screening network was established [26]. By sharing information, internet hospitals can help recognize individuals with a higher probability of being infected. Combined with the offline screening conducted by the epidemiologic investigation organizations and the community offices, online consultations and follow-ups may reduce the risk of missed screenings. Meanwhile, self-isolation [27,28] was required for all symptomatic counselees and epidemiologically close contacts [29]. By identifying these counselees, the internet hospitals can facilitate offline supervision for potentially undocumented cases and promote social distancing [30].

In the aspect of auxiliary functions, internet hospitals provide basic medical support to the public during the epidemic. Affected by the outbreak, numerous nonemergency outpatient departments were closed in Chinese hospitals, causing most offline clinics to be unavailable for the public. Through internet hospitals, the patients could keep in touch with their attending doctors. For those who had new mild symptoms, the doctors could give professional advice on self-management of care and treatment. For those in severe conditions, online doctors may guide them to visit offline hospitals as soon as possible in case of deterioration. Moreover, during the period of individual self-isolation, internet hospitals may join the social capital, improving quality of life by reducing anxiety and stress [31].

### Limitations of Internet Hospitals

Fully understanding the positive role played by internet hospitals during the epidemic, we should also realize their limitations. First, online consultation is an indirect way of communication. Due to the lack of information such as physical and auxiliary examinations, online doctors may only give rough medical advice for primary care patients. Second, most of the internet hospitals in China currently offer only passive order-based services to the public. In terms of epidemic screening, it is necessary to cooperate with offline approaches to make better use of the internet hospitals’ online advantages. Third, the audience of internet hospitals has not yet covered the whole population due to the difference in public acceptance, which can be reflected by the unbalanced distribution of the counselees’ ages. Furthermore, the accessibility of the internet is another limitation of internet hospitals.

### Directions for Future Efforts

To make better use of internet hospitals during the epidemic, more efforts are needed, such as recruiting more doctors, especially psychologists [32] and pediatricians, to join the online services while ensuring that each attending doctor has mastered the latest epidemic prevention and control knowledge; discovering the public’s needs in a timely manner to adjust our response strategies; improving the usability of the internet hospital apps; strengthening the propaganda to expand the user base; and cooperating with the communities and the CDC to improve joint control and prevention mechanisms [33]. Meanwhile, a more standardized consultation service guideline is needed, enacting different response strategies according to different counselee types. In our study, online education and propaganda were needed for all counselees; self-isolation guidance was needed for all suspicious and exposed counselees; psychological intervention was badly needed for hypochondriacal counselees; and offline visit recommendations were essential when the counselees were in severe conditions. The data of the suspicious counselees and their corresponding risk levels should be highlighted and shared through the joint control and prevention system run by the official agencies for further interventions.

### Limitations and Conclusions

This study has some limitations. First, our data was collected outside of the Hubei province. In relatively low-prevalence areas, the hypochondria and panic rate might be underestimated. Meanwhile, the characteristics of the counselees were collected according to their description rather than standardized questionnaires. Some symptoms might be neglected by the counselees and, thus, also underestimated. Second, there are other forms of online consultation services, including paid services held by general public hospitals or private companies [34], which need further estimation. Moreover, the follow-up information after the online consultations were unavailable due to privacy reasons. More longitudinal studies are required to evaluate the counselees’ compliance to the online services. Further studies may focus on how to optimize the online services during the epidemic and expand internet hospitals’ beneficial influences on the public [35]. As COVID-19 has quickly became a global threat, all countries should consider a combination of response measures with telemedicine platforms involved [36]. According to our results, remote medical services are badly needed for the panicked public. Internet hospitals can make targeted and tailored medical interventions for various types of counselees and help prevent and control the epidemic of COVID-19 in China.

## Appendix

Multimedia Appendix 1 The complete results of logistic regression models.

## Abbreviations

CDC: Centers for Disease Control and Prevention
COVID-19: coronavirus disease
OR: odds ratio
