# Supplementary material for: Internet Hospitals Help Prevent and Control the Epidemic of COVID-19 in China: Multicenter User Profiling Study
Source: J Med Internet Res. 2020 Apr 14;22(4):e18908. doi: 10.2196/18908 (PMC7159055; doi:10.2196/18908)
Supplement: Multimedia Appendix 1 [file jmir_v22i4e18908_app1.doc]

Supplementary table 1: univariate logistic regression for hypochondriacal suspicion

| Predictor | Number of Consultants | OR (95%CI) | P Value |
| --- | --- | --- | --- |
| Epidemiological exposure | 259 | 8.107(5.718,11.495) | <0.001 |
| Adulthood | 4056 | 2.504(2.127,2.949) | <0.001 |
| Fever(≥37.5℃) | 2021 | 0.544(0.484,0.612) | <0.001 |
| Unrelated symptoms | 692 | 1.662(1.414,1.954) | <0.001 |
| Severe illness | 190 | 0.428(0.308,0.596) | <0.001 |
| Shortness of breath | 365 | 1.712(1.381,2.123) | <0.001 |
| Mild fever(<37.5℃) | 878 | 1.272(1.099,1.473) | 0.001 |
| Cough | 2118 | 0.835(0.745,0.936) | 0.002 |
| Fatigue | 415 | 1.361(1.113,1.664) | 0.003 |
| Diarrhea | 350 | 1.325(1.066,1.647) | 0.011 |
| Myalgia | 301 | 1.317(1.043,1.663) | 0.021 |
| Expectoration | 752 | 0.889(0.759,1.040) | 0.142 |
| Re-attendance | 286 | 0.859(0.673,1.095) | 0.219 |
| Sex(male) | 2031 | 0.941(0.839,1.055) | 0.294 |
| Headache | 443 | 1.070(0.880,1.302) | 0.496 |
| Nasal congestion | 981 | 1.046(0.909,1.204) | 0.531 |
| Nausea and vomiting | 282 | 1.042(0.819,1.327) | 0.736 |
| Sore throat | 735 | 1.007(0.860,1.179) | 0.929 |
| Chills | 16 | 0.987(0.367,2.655) | 0.980 |

Supplementary table 2: multivariate logistic regression for hypochondriacal suspicion

| Predictor | Number of Consultants | OR (95%CI) | P Value |
| --- | --- | --- | --- |
| Epidemiological exposure | 259 | 7.568(5.316,10.773) | <0.001 |
| Adulthood | 4056 | 1.816(1.520,2.169) | <0.001 |
| Fever(≥37.5℃) | 2021 | 0.655(0.568,0.754) | <0.001 |
| Unrelated symptoms | 692 | 1.509(1.273,1.789) | <0.001 |
| Shortness of breath | 365 | 1.440(1.150,1.803) | 0.001 |
| Cough | 2118 | 0.832(0.734,0.943) | 0.004 |
| Diarrhea | 350 | 1.272(1.011,1.599) | 0.040 |
| Fatigue | 415 | 1.222(0.991,1.508) | 0.061 |
| Severe illness | 190 | 0.749(0.529,1.059) | 0.102 |
| Myalgia | 301 | 1.222(0.959,1.559) | 0.105 |
| Mild fever(<37.5℃) | 878 | 0.944(0.798,1.117) | 0.502 |

Supplementary table 3: univariate logistic regression for offline-visit motivation

| Predictor | Number of Consultants | OR (95%CI) | P Value |
| --- | --- | --- | --- |
| Hypochondriacal suspicion | 2165 | 4.428(3.764,5.210) | <0.001 |
| Epidemiological exposure | 259 | 2.233(1.698,2.935) | <0.001 |
| Severe illness | 190 | 1.852(1.337,2.566) | <0.001 |
| Fever | 2021 | 1.304(1.125,1.511) | <0.001 |
| Re-attendance | 286 | 0.591(0.408,0.857) | 0.005 |
| Sex(male) | 2031 | 0.853(0.734,0.992) | 0.039 |
| Shortness of breath | 365 | 1.314(1.012,1.705) | 0.040 |
| Mild fever(<37.5℃) | 878 | 1.167(0.969,1.405) | 0.103 |
| Cough | 2118 | 1.104(0.952,1.279) | 0.190 |
| Diarrhea | 350 | 0.839(0.622,1.132) | 0.251 |
| Adulthood | 4056 | 0.914(0.756,1.105) | 0.354 |
| Nausea and vomiting | 282 | 1.136(0.838,1.540) | 0.411 |
| Unrelated symptoms | 692 | 0.939(0.758,1.162) | 0.562 |
| Chills | 16 | 0.664(0.151,2.927) | 0.589 |
| Nasal congestion | 981 | 0.952(0.792,0.146) | 0.606 |
| Expectoration | 752 | 1.044(0.853,1.277) | 0.679 |
| Sore throat | 735 | 0.967(0.786,1.190) | 0.753 |
| Myalgia | 301 | 1.019(0.752,1.380) | 0.906 |
| Headache | 443 | 1.011(0.783,1.305) | 0.933 |
| Fatigue | 415 | 0.993(0.762,1.293) | 0.957 |

Supplementary table 4: multivariate logistic regression for offline-visit motivation

| Predictor | Number of Consultants | OR (95%CI) | P Value |
| --- | --- | --- | --- |
| Hypochondriacal suspicion | 2165 | 4.826(4.068,5.724) | <0.001 |
| Severe illness | 190 | 2.303(1.608,3.300) | <0.001 |
| Fever(≥37.5℃) | 2021 | 1.660(1.410,1.953) | <0.001 |
| Re-attendance | 286 | 0.545(0.371,0.801) | 0.002 |
| Epidemiological exposure | 259 | 1.440(1.082,1.918) | 0.012 |
| Sex(male) | 2031 | 0.854(0.730,1.001) | 0.051 |
| Shortness of breath | 365 | 1.253(0.951,1.651) | 0.109 |
